# Alzheimer's disease—Biomarkers, clinical evaluation or both?

**DOI:** 10.1111/jnp.12401

**Published:** 2024-11-14

**Authors:** Joel Simrén, Nicholas J. Ashton, Marc Suárez‐Calvet, Henrik Zetterberg

**Affiliations:** ^1^ Department of Psychiatry and Neurochemistry Institute of Neuroscience and Physiology, The Sahlgrenska Academy at the University of Gothenburg Gothenburg Sweden; ^2^ Clinical Neurochemistry Laboratory Sahlgrenska University Hospital Mölndal Sweden; ^3^ Banner Alzheimer's Institute Phoenix Arizona USA; ^4^ Banner Sun Health Research Institute Sun City Arizona USA; ^5^ Barcelonaβeta Brain Research Center (BBRC) Pasqual Maragall Foundation Barcelona Spain; ^6^ Hospital del Mar Research Institute Barcelona Spain; ^7^ Servei de Neurologia Hospital del Mar Barcelona Spain; ^8^ Department of Neurodegenerative Disease Institute of Neurology, UCL London UK; ^9^ UK Dementia Research Institute, UCL London UK; ^10^ Hong Kong Center for Neurodegenerative Diseases Clear Water Bay Hong Kong China; ^11^ Wisconsin Alzheimer's Disease Research Center University of Wisconsin School of Medicine and Public Health Madison Wisconsin USA

Recent developments in fluid and imaging biomarkers that reflect the key pathological hallmarks of Alzheimer's disease (AD)—deposits of extracellular amyloid‐β (Aβ) and intracellular tau proteins—have transformed the perception of the disease in living individuals from a clinical syndrome to a biological continuum that begins prior to the onset of symptoms (Scheltens et al., 2021). Over the past two decades, biomarker research has revealed that Aβ deposition and abnormal tau metabolism begin years before symptoms appear, following a predictable sequence of biological changes (Bateman et al., 2012; Villemagne et al., 2013). This suggests a prolonged preclinical phase of the disease. Biomarkers, which have greatly expanded our understanding of disease progression, are now routinely applied in clinical settings. These include Food and Drug Administration (FDA)‐approved positron emission tomography (PET) imaging agents of Aβ plaques and tau aggregates, cerebrospinal fluid (CSF) measures of Aβ and phosphorylated tau (p‐tau), and soon, plasma measures of tau forms phosphorylated at amino acid 217 (p‐tau217).

As AD neuropathology is the defining hallmark of the disease (Hyman et al., 2012), as well as being the target of emerging treatments, recently approved in some countries (Cummings et al., 2023), it is reasoned that biomarkers that directly reflect these changes should be the defining features of the disease. This view was formally articulated in the recent publication of novel Alzheimer's Association diagnostic and staging criteria for AD, which suggest that the disease can be diagnosed when a so‐called ‘Core 1’ biomarker of Aβ proteinopathy or phosphorylated and secreted tau is abnormal, resulting in a purely biological definition of the disease (Jack et al., 2024). In prior years, studies have shown that PET detect Aβ (Clark et al., 2012) and tau (Fleisher et al., 2020) neuropathology with high (~90%) accuracy. CSF tests of Aβ42/40 and Aβ42/p‐tau (Janelidze et al., 2017) have been validated against amyloid PET with similar accuracy, and subsequently also neuropathology (Mattsson‐Carlgren et al., 2022). Over the past 5 years, an expanding body of research indicates that plasma p‐tau217 can detect Aβ pathology with high accuracy (Ashton et al., 2023, 2024; Schindler et al., 2024), which will improve the access to biological AD diagnoses in clinical settings beyond what health care systems are currently scaled to accommodate.

The recently published diagnostic and staging criteria (Jack et al., 2024) are a development of the criteria published by the National Institute of Aging and Alzheimer's Association (NIA‐AA) in 2018, which aimed to establish a common language for further research in the biological evolution of AD and its relation to resulting symptomatology (Jack et al., 2018). As in that document, a clinical staging scheme is added as an adjunct to the disease definition in the recent criteria (Jack et al., 2024). The recent criteria also added a biological staging scheme, suggesting that tau PET (‘Core 2 biomarker’; including promising albeit explorative fluid biomarkers of tau aggregates; Horie et al., 2023) is used in conjunction with Aβ PET to biologically stage the disease, due to the closer relationship of tau aggregates with symptomatology (Ossenkoppele et al., 2018).

Another key development that preceded these criteria was the recent full regulatory approval of lecanemab (van Dyck et al., 2023) and donanemab (Sims et al., 2023) by the FDA, and now several other regulatory bodies in other parts of the world as well. These trials relied on the careful use of biomarker‐based inclusion, ensuring that the individuals had the pathology targeted by the treatment (i.e. Aβ pathology). This was not the case in some earlier failed trials, where AD dementia was defined clinically, meaning that the diagnosis was agnostic to biomarker status. In one of these trials, a significant proportion of study participants were found to be Aβ negative (Salloway et al., 2014), and thus likely misdiagnosed. In other words, to successfully treat the biology of a disease, the definition of the disease must be based on its underlying biology. Furthermore, biomarkers such as plasma p‐tau217 and glial fibrillary acidic protein (GFAP; an astroglial protein reflecting glial actviation) showed a response to anti‐Aβ drugs in clinical trials, suggesting that they could be used as target engagement biomarkers (Pontecorvo et al., 2022; Sims et al., 2023).

Further supporting this, the recent progress in CSF and imaging biomarkers, as well as molecular neuropathology, has enabled the field to gain greater knowledge on the relationship between neuropsychological measures, symptomatology and biomarker/neuropathological findings. These studies have found that the classical amnestic syndrome usually associated with AD pathology may instead be due to limbic‐predominant age‐related TDP‐43 encephalopathy (LATE) (Nelson et al., 2019) in a significant proportion of cases, particularly in the oldest old patients. Conversely, syndromes such as primary progressive aphasia (Bergeron et al., 2018), behavioural/dysexecutive syndromes (Ossenkoppele et al., 2015), corticobasal syndrome (Lee et al., 2011) and posterior cortical atrophy (Alladi et al., 2007) may be also associated with underlying AD pathology.

It is well known that individuals may present with multiple pathologies (Robinson et al., 2018), have different cognitive resilience to pathologies (Stern, 2012), have genetic modifiers of disease progression (Van Cauwenberghe et al., 2016), or have symptomatology not typically associated with AD (e.g. fluctuations in cognitive symptoms, being common in dementia with Lewy bodies), which can be reflected in dissociation between clinical stage and biological stage (Jack et al., 2024). Once again, however, it does not mean that the disease is *not* present if a positive biomarker is not consistent with the clinical manifestations. On the other hand, careful clinical judgement is needed to determine what pathology most likely explains the symptomatology.

Critics of the biological definition of AD have expressed that these new criteria would enable detecting asymptomatic individuals with AD pathology, even though it is not certain whether they will eventually develop symptoms due to the disease. Nevertheless, the Alzheimer's Association criteria emphasizes that AD *can*, but *should not*, currently be diagnosed in individuals without cognitive symptoms (clinical stage 1) (Jack et al., 2024), with the same reasoning being applicable in most individuals with subjective cognitive complaints (i.e. no objective impairment; clinical stage 2), as there are no approved treatments for this group, and the disease prevalence in these cases is low, and hence, the positive predictive values are lower, resulting in higher falsely positive results and unnecessary investigations and anxiety (Hansson & Jack, 2024). This may change if ongoing trials in preclinical AD are successful (Rafii et al., 2023), leading to a scenario comparable with cardiovascular disease (e.g. treatment of hypertension or lipid lowering) or type 2 diabetes (treatment of asymptomatic hyperglycemia). In the absence of such treatments, we believe that diagnosing AD with biomarkers should currently be conducted only in symptomatic individuals, in tandem with a careful clinical evaluation, where there is higher pre‐test probability for the disease, and actionable consequences (e.g. symptomatic and in some countries disease‐modifying, treatments). Specific workflows and scenario‐based guidelines of how and when AD should be diagnosed are under development.

Future research of great importance to increase the confidence in that AD is the cause of symptoms for a certain patient include validation of fluid biomarkers that reflect tau aggregates (i.e. provide similar information as tau PET). Promising results have been shown for CSF measures of microtubule‐binding region (MTBR) tau (Horie et al., 2023; Salvado et al., 2024) and CSF or plasma p‐tau at amino acid 205 (p‐tau205) (Gobom et al., 2022; Lantero‐Rodriguez et al., 2024; Montoliu‐Gaya, Alosco, et al., 2023; Montoliu‐Gaya, Benedet, et al., 2023), which better reflect tau aggregates than markers reflecting Aβ. Yet, if such biofluid measures can accurately reflect stage at the individual level is yet to be proven.

Further, the possibility of testing patients outside highly specialized contexts with plasma biomarkers require large education efforts for general practitioners on how to interpret and communicate biomarker test results in different patient categories, which require close collaboration between expert centres with primary care providers. Emphasis should be put on how to interpret biomarker results around predefined cut‐offs, as biomarkers are continuous in their nature, on which a dichotomous categorization is superimposed (Jack et al., 2024). Therefore, one should be careful to interpret these values, for which strategies are currently developed, with an intermediate range, or grey zone, being one example (Brum et al., 2022). As with any clinical chemistry test, the results should be interpreted in a complete clinical context not to cause more harm than good. For example, in a slightly depressed patient with cognitive problems and positive AD biomarkers it will be very important to consider the possibility that it may be the depression that causes the symptoms, and that the biomarker positivity may reflect preclinical pathology that may or may not bother the patient in the coming 10 years. Whenever in doubt, clinical judgement, and careful follow‐up with the potential to reconsider a diagnosis will be as important as ever.

We view the changing definition of AD to be an important step towards effective therapies, which target disease biology, although careful evaluation of symptomatology is crucial both as a gatekeeping function to ensure that an AD diagnosis is given only to relevant patient populations, and to inform on the likelihood that AD is contributing to the symptoms. Integrating diagnostic biomarkers with clinical and biological staging provides a foundation to make meaningful diagnoses based on clinical judgement that are consistent with the current understanding of the evolution of AD as a biological entity.

## AUTHOR CONTRIBUTIONS

**Joel Simrén:** Writing – original draft; writing – review and editing. **Nicholas J. Ashton:** Writing – original draft; writing – review and editing. **Marc Suárez‐Calvet:** Writing – review and editing; writing – original draft. **Henrik Zetterberg:** Writing – original draft; writing – review and editing.

## CONFLICT OF INTEREST STATEMENT

JS reports no conflicts of interest. NJA has served at scientific advisory boards and/or as a consultant for Alamar Biosciences, Biogen, TauRx, TargetALS, Quanterix and has given lectures sponsored by BioArctic, Biogen, Lilly ad VJDementia. MS‐C has received in the past 36mo consultancy/speaker fees (paid to the institution) from by Almirall, Eli Lilly, Novo Nordisk, and Roche Diagnostics. He has received consultancy fees or served on advisory boards (paid to the institution) of Eli Lilly, Grifols and Roche Diagnostics. He was granted a project and is a site investigator of a clinical trial (funded to the institution) by Roche Diagnostics. In‐kind support for research (to the institution) was received from ADx Neurosciences, Alamar Biosciences, ALZPath, Avid Radiopharmaceuticals, Eli Lilly, Fujirebio, Janssen Research & Development, Meso Scale Discovery, and Roche Diagnostics; MS‐C did not receive any personal compensation from these organizations or any other for‐profit organization. HZ has served at scientific advisory boards and/or as a consultant for Abbvie, Acumen, Alector, Alzinova, ALZpath, Amylyx, Annexon, Apellis, Artery Therapeutics, AZTherapies, Cognito Therapeutics, CogRx, Denali, Eisai, LabCorp, Merry Life, Nervgen, Novo Nordisk, Optoceutics, Passage Bio, Pinteon Therapeutics, Prothena, Quanterix, Red Abbey Labs, reMYND, Roche, Samumed, Siemens Healthineers, Triplet Therapeutics, and Wave, has given lectures sponsored by Alzecure, BioArctic, Biogen, Cellectricon, Fujirebio, Lilly, Novo Nordisk, Roche, and WebMD, and is a co‐founder of Brain Biomarker Solutions in Gothenburg AB (BBS), which is a part of the GU Ventures Incubator Program (outside submitted work).

## Data Availability

There is no data.
